# Research on Gait Planning for Wind Turbine Blade Climbing Robots Based on Variable-Cell Mechanisms

**DOI:** 10.3390/s26020547

**Published:** 2026-01-13

**Authors:** Hao Lu, Guanyu Wang, Wei Zhang, Mingyang Shao, Xiaohua Shi

**Affiliations:** 1 School of Electronic Information and Automation, Tianjin University of Science and Technology, Tianjin 300450, China; 99013921@tust.edu.cn (H.L.); wangguanyu-a@aliyun.com (G.W.); 18360783536@163.com (W.Z.); 2 School of Mechanical Engineering, Yanshan University, Qinhuangdao 066004, China; 15630120871@163.com

**Keywords:** climbing robot, metamorphic mechanism, gait planning, static stability margin analysis

## Abstract

To address the complex surface curvature, massive dimensions, and variable pitch angles of wind turbine blades, this paper proposes a climbing robot design based on a variable-cell mechanism. By dynamically adjusting the support span and body posture, the robot adapts to the geometric features of different blade regions, enabling stable and efficient non-destructive inspection operations. Two reconfigurable configurations—a planar quadrilateral and a regular hexagon—are proposed based on the geometric characteristics of different blade regions. The configuration switching conditions and multi-leg cooperative control mechanisms are investigated. Through static stability margin analysis, the stable gait space and maximum stride length for each configuration are determined, optimizing the robot’s motion performance on surfaces with varying curvature. Simulation and experimental results demonstrate that the proposed multi-configuration gait planning strategy exhibits excellent adaptability and climbing stability across segments of varying curvature. This provides a theoretical foundation and methodological support for the engineering application of robots in wind turbine blade maintenance.

## 1. Introduction

The urgent global demand for clean energy has driven the large-scale deployment of wind power generation. As the core component of wind turbines, the operational condition of wind turbine blades directly impacts power generation efficiency and structural safety. Cieslak et al. developed a specialized inspection robot system tailored to wind turbine maintenance needs, highlighting that long-term exposure to harsh environments can cause surface wear, cracks, and other damage to blades. Failure to detect such issues promptly may lead to safety incidents [1]. With the evolution of robotics technology, it has emerged as an effective solution for automated inspection of complex surfaces. Hernando et al. demonstrated the engineering feasibility of robotic technology in infrastructure inspection through their modular climbing robot Romerin [2]. Wang et al. further introduced neural networks into path planning for wind turbine blade inspection robots, exploring new avenues for intelligent inspection [3]. However, traditional fixed-configuration robots struggle to maintain stable adhesion on complex curved surfaces, varying curvatures, and surface obstacles, limiting their mobility. Metamorphic mechanisms overcome these challenges through active reconfiguration: adhesion modules dynamically conform to curved surfaces to ensure sealing integrity, while structural state transitions enable obstacle clearance, offering a theoretically viable technical solution.

Despite significant progress in the kinematic theory of variable-cell mechanisms, their engineering applications and gait planning research in the field of climbing robots remain insufficient. Kang et al. revealed the higher-order bifurcation characteristics of Schatz-inspired variable-cell mechanisms based on spiral theory, laying a theoretical foundation for mechanism innovation [4]; Shi et al. introduced a closed-loop 10R-Folding mechanism into the torso of variable-cell robots, expanding the configuration space [5]; Chai et al. constructed six novel 6R variable-cell mechanisms using a triple-tandem Bennett linkage, enriching mechanism topology [6]; Jia et al. systematically synthesized scissor-type variable-cell mechanisms based on different link pairs, offering diverse design options [7]; Wang et al. investigated the reconfigurability of an integrated 8R motion-topology variable-cell mechanism and its derivatives, inspired by origami [8]; Zhou et al. conducted an in-depth analysis of impact dynamics effects during configuration transitions in variable-cell mechanisms [9], providing critical insights for dynamic design. While these studies primarily focus on configuration synthesis and kinematic analysis, systematic approaches remain elusive for practical applications like blade inspection. Specifically, coordinating mechanism reconfiguration with robotic motion control—particularly planning continuous, stable gaits across configuration states—has yet to be systematically addressed.

Multi-legged configurations represent the mainstream solution for climbing robots, where gait planning directly determines environmental adaptability and motion performance. Existing research has established multiple methodological frameworks: Valouch and Faigl proposed a caterpillar-inspired gait-free planning strategy to simplify multi-legged coordination [10]; Winkler et al. achieved gait and trajectory optimization for leg systems through phase-based end-effector trajectory parameterization [11]; Neunert et al. integrated contact dynamics into trajectory optimization, enabling automatic gait discovery for quadrupedal robots [12]. At the control framework level, Bjelonic et al. developed full-body model predictive control and online gait sequence generation for wheel-legged robots [13]; He and Gao systematically reviewed kinematic, drive, perception, and control technologies for high-dynamic multi-legged robots [14]; Chong et al. constructed a universal motion control framework for multi-legged actuators, enhancing cross-platform adaptability [15]. For complex environments, Belter investigated integrating natural terrain semantics into motion planning [16] and proposed efficient path constraint evaluation methods [17]; Buchanan et al. achieved perceptive full-body planning for multi-legged robots in confined spaces [18]; Tennakoon et al. designed a step-probing strategy to mitigate risks on collapsible terrain [19]. In terms of stability and dynamics analysis, Liu et al. performed precise calculations of static foot forces during the three-legged gait of heavy-duty hexapods [20]; Roy and Pratihar established a dynamic model and energy consumption analysis framework for realistic hexapods [21]; Goswami’s Foot Rotation Index (FRI) point theory offered a novel perspective on multi-legged stability criteria [22]. Innovations in intelligent gait generation methods continue: Wang et al. enhanced central pattern generator (CPG)-based hexapod gait planning by integrating feedback signals [23]; Liu et al. achieved fault-tolerant tripod gait design for hexapods [24]; Tsounis et al. completed quadruped gait planning and control using deep reinforcement learning [25]. Xu et al. analyzed quadruped gait characteristics based on the variable cell concept [26]; Tian and Gao enhanced the locomotion efficiency of a hexapod robot on irregular terrain by integrating foot placement selection with whole-body optimization planning [27]; Wang et al. systematically analyzed typical motion patterns of a symmetric hexapod robot [28]; Zha et al. designed a free gait controller for a heavy-duty hexapod robot [29]; Grzelczyk and Awrejcewicz investigated modeling and control of an eight-legged robot driven by multiple gait generators [30]; Go et al. theoretically explored the navigability limits of multi-legged robots [31].

In recent years, research on specialized configurations and scenarios has proliferated: Xu et al. proposed deep reinforcement learning-based adaptive motion planning for spherical multi-telescopic-leg robots [32]; Chen et al. achieved autonomous gait switching for hexapod robots in Mars’ multi-terrain environments [33]; Gong et al. investigated the agile planar transition capability of a hexapod climbing robot [34]; Nadan et al. developed LORIS, a lightweight free-climbing robot for extreme terrain exploration [35]; Fang et al. significantly enhanced the performance of legged wall-climbing robots through a dynamic contact integration model [36]; Lou et al. systematically reviewed the current status and trends in wall-climbing robot research [37]; Chen et al. designed and optimized the structure of an adaptive soft-adhesive bionic climbing robot [38].

However, most of the above achievements focus on robots with fixed configurations. Even when gait switching is involved [33,34], the reconstruction process of the mechanism topology itself is not considered. For variable-cell robots, configuration transitions instantaneously alter the supporting polygon topology, center-of-mass distribution, and kinematic constraints. Traditional gait planning methods designed for single configurations cannot be directly applied. Existing research has yet to establish a coordinated planning theory for cross-configuration motion in variable-cell robots, particularly lacking adaptive multi-configuration gait generation methods that simultaneously address blade surface adaptation, obstacle-crossing requirements, and motion stability.

To address the aforementioned research gap, this paper designs a variable-cell climbing robot for wind turbine blade inspection, focusing on its multi-configuration adaptive gait planning method. Through systematic analysis of the variable-cell mechanism’s configurations, two crawling modes are identified, and the switching conditions between different configurations along with leg coordination mechanisms are investigated. Subsequently, dedicated gait schemes are designed for each configuration, incorporating static stability margin theory to optimize the gait space. Ultimately, simulations and prototype experiments validated the robot’s adhesion reliability and gait stability across multiple configurations, providing a theoretical foundation and methodological support for its engineering application in wind turbine blade maintenance.

The remainder of this paper is organized as follows: Section 2 briefly introduces the robot’s mechanism design and proposes three configuration modes. Section 3 defines the transition conditions between configurations. Section 4 details gait generation and static stability margin analysis for the climbing robot in both modes. Section 5 experimentally verifies the load-bearing capability and gait stability of the prototype robot under different configurations, demonstrating its stability. Finally, conclusions are presented in Section 6.

## 2. Structural Design and Configuration Changes

**Figure 1:**
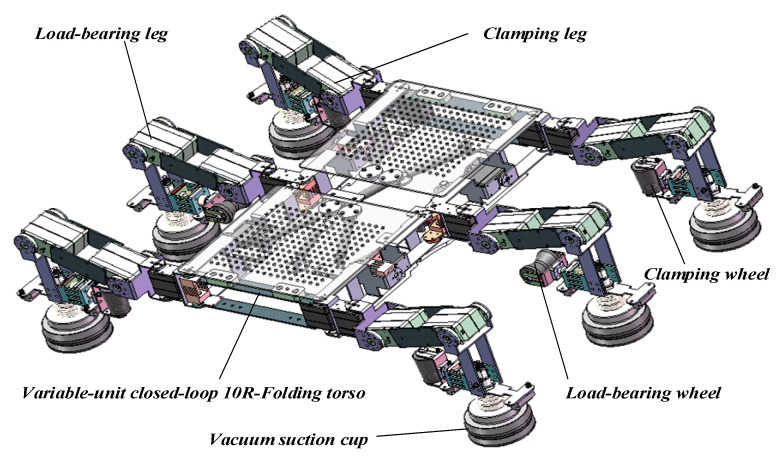
Robot 3D prototype model.

**Figure 2:**
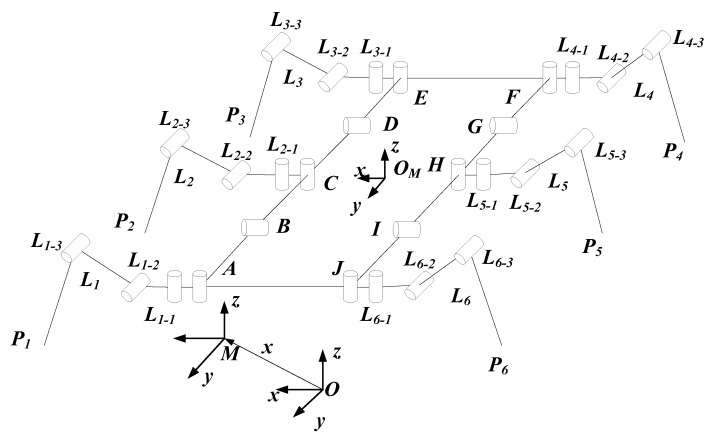
Schematic diagram of robot mechanism.

The three-dimensional prototype model and simplified mechanical diagram of the climbing robot designed in this paper are shown in Figure 1 and Figure 2. The robot’s body employs a 10R-Folding torso with variable-cell closed-loop folding and deformation capabilities. Its legs utilize a three-degree-of-freedom integrated wheel-leg composite mechanism, comprising six legs: four gripping legs and two load-bearing legs. Furthermore, the robot employs vacuum suction technology to achieve a simple, compact Configuration with reduced self-weight. Through optimized design, it meets the adaptive conforming requirements for blade surfaces, effectively controlling overall weight while ensuring suction performance. Nitrile rubber, selected for its excellent wear resistance and tear strength, serves as the material for the vacuum suction cups.

For simplicity, the following Parameters are defined: ***A***, ***E***, ***F*** denote corner joint servo motors; ***B***, ***D***, ***G***, ***I*** denote two-end swing joint servo motors; ***J*** denotes a passive rotating joint; ***C***, ***H*** denote motor connection components; ***AB***, ***DE***, ***EF***, ***FG***, ***IJ***, ***JA*** denote carbon fiber connecting plates; ***L*_1_**, ***L*_3_**, ***L*_4_**, ***L*_6_** denote clamping legs; ***L*_2_**, ***L*_5_** denote load-bearing legs. ***P*_1_**, ***P*_2_**, ***P*_3_**, ***P*_4_**, ***P*_5_**, ***P*_6_** denote toe suction cups; ***O-xyz***, ***M-xyz***, ***O_M_-xyz*** represent the world coordinate system, body projection coordinate system, and body coordinate system, respectively. ***O-xyz*** is rigidly fixed to the wind turbine blade surface, while ***O_M_-xyz*** is defined at the body’s geometric center with its ***y-axis*** passing through the midpoint of two short rods and pointing forward. ***M-xyz*** is the projection of ***O_M_-xyz*** onto the ***O-xy*** plane, maintaining the same ***z-axis*** direction. The joint functional groups of the wind turbine blade climbing robot are shown in Table 1.

**Figure 3:**
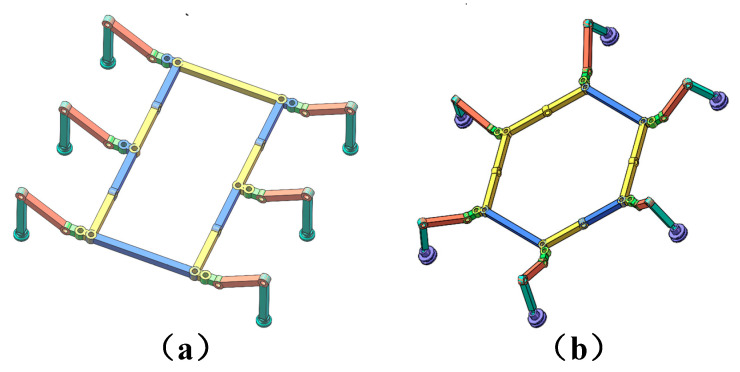
Two structural state models: (**a**) ***Configuration I***; (**b**) ***Configuration II***.

This paper provides a flexible structural foundation for gait planning through the design of a variable-cell mechanism. Two configurations are proposed to adapt to terrain variations across different regions of the wind turbine blade. Subsequent sections will detail the configuration transition process and gait planning for both configurations. Schematic diagrams of the two configurations are shown in Figure 3.

1. ***Configuration I*** is a planar quadrilateral configuration. Serving as the initial configuration for climbing robots, it enables flexible transitions to the other two configurations and functions as a stable state for the robot’s climbing progression;
2. ***Configuration II*** is a regular hexagonal configuration. Compared to Configuration I, each leg possesses a greater range of motion, with the basal segment of each leg capable of rotating 240°. This configuration offers higher turning efficiency and enables movement along all six orientations of vertical edges even without turning.

The configuration and motion mode of the morphing climbing robot are adjusted in response to environmental changes. In-depth research is required on configuration switching and leg coordination to define switching conditions, ensuring the robot’s center of gravity remains within a safe support range throughout the transition. This provides stability assurance for gait planning.

**Figure 4:**
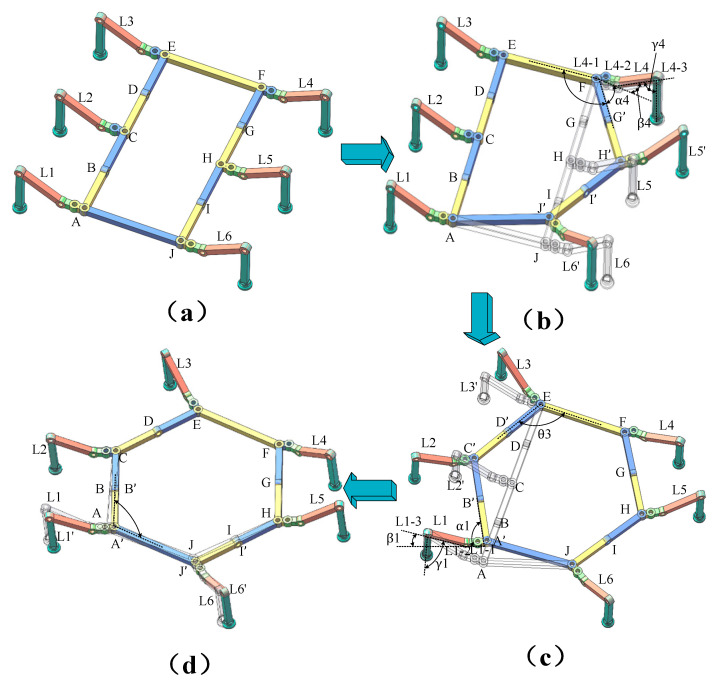
Schematic diagram of the transition from ***Configuration*** ***I*** to ***Configuration*** ***II***: (**a**) ***Configuration*** ***I***; (**b**) ***Transition*** ***State I***; (**c**) ***Transition*** ***State II***; (**d**) ***Configuration*** ***II***.

Transitioning from ***Configuration I*** to ***Configuration II*** involves two intermediate configurations. During switching, the robot’s legs maintain adhesion to the wind turbine blade surface via vacuum suction cups to prevent falling. The specific process is as follows: ***L*_1_**, ***L*_2_**, ***L*_3_**, and ***L*_4_** maintain adhesion while ***L*_5_** and ***L*_6_** lift. The motor at joint ***F*** rotates to form angle ***EFH*** of 120°, causing the linkage to transform. Simultaneously, the coxal joint ***L*_4*-*1_** is rotated to ***α*_4_**, while the femoral joint ***L*_4*-*2_** is rotated to ***β*_4_**, and the tibial joint ***L*_4*-*3_** is rotated to ***γ*_4_**, completing ***Transition state I***. Next, ***L*_1_**, ***L*_4_**, ***L*_5_**, and ***L*_6_** maintain adhesion while ***L*_2_** and ***L*_3_** lift. The motor at joint ***E*** rotates to form angle ***CEF*** of 120°, changing the linkage configuration. The coxal joint ***L*_1*-*1_** is rotated to ***α*_1_**, while the femoral joint ***L*_1*-*2_** is rotated to ***β*_1_**, and the tibial joint ***L*_1*-*3_** is rotated to ***γ*_1_**, completing ***Transition state II***; Finally, ***L*_2_**, ***L*****_3_**, ***L*_4_**, and ***L*_5_** maintain adhesion while ***L*_1_** and ***L*_6_** lift. The motor at joint ***A*** rotates to form angle ***JAC*** of 120°, causing the linkage to transform. The joint motor at ***L*****_2_** remains unchanged, completing the switch to ***Configuration II***. The switching process diagram is shown in Figure 4.

## 3. Gait Generation

Gait planning directly impacts a robot’s motion efficiency and stability. As a key technology in motion control, multi-legged robot gait planning centers on coordinating limb movement phases with optimized ground contact timing. Based on the aforementioned mechanism design and configuration switching, this paper focuses on gait planning methods for robots operating in two configurations. It designs a triangular gait and employs static stability margin theory to quantitatively analyze gait parameters, thereby meeting the demands of efficient climbing on complex surfaces like wind turbine blades.

### 3.1. Configuration I Gait Generation

**Figure 5:**
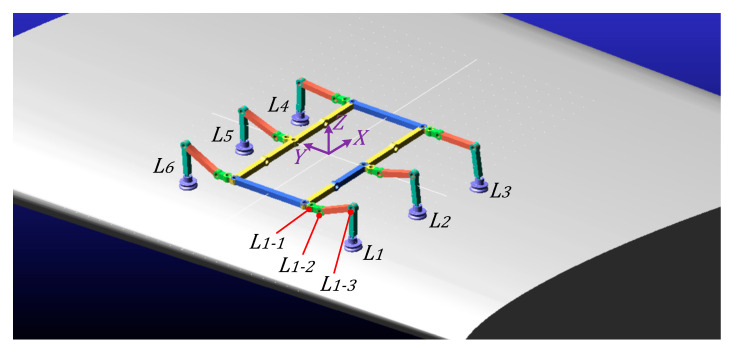
***Configuration I*** gait coordinate diagram: ***L*_1*-*1_** denotes the basal segment motor, ***L*_1*-*2_** denotes the femoral segment motor, and ***L*_1*-*3_** denotes the tibial segment motor.

The initial state of the robot placed on the blade is shown in Figure 5, with each leg’s basal segment forming a 90° angle with the torso. During the preparation phase of the triangular gait in ***Configuration*** ***I***, each leg must be swung to the initial state of the triangular gait: ***L*_1_**, ***L*****_3_**, and ***L*****_5_** swing forward 30°, forming a 120° angle with the body; ***L*_2_**, ***L*_4_**, and ***L*_6_** swing backward 30°, forming a 60° angle with the body. The triangular gait consists of two groups: ***L*_1_**, ***L*****_3_**, and ***L*_5_** form one group, while ***L*_2_**, ***L*_4_**, and ***L*_6_** form another. ***L*****_6_** swing backward 30°, forming a 60° angle with the body. ***L*_1_**, ***L*_3_**, and ***L*_5_** form one leg group, while ***L*_2_**, ***L*****_4_**, and ***L*_6_** form another. These leg groups alternate between swing and stance phases during gait, with each swing angle measuring 60°. During the swing phase, the swing leg group lifts as the base joint motor rotates, propelling the swing leg group forward. During the support phase, the suction cups at the ends of the support leg group firmly adhere to the blade surface. Rotation of the base joint motor drives the torso forward, shifting the center of gravity.

**Figure 6:**
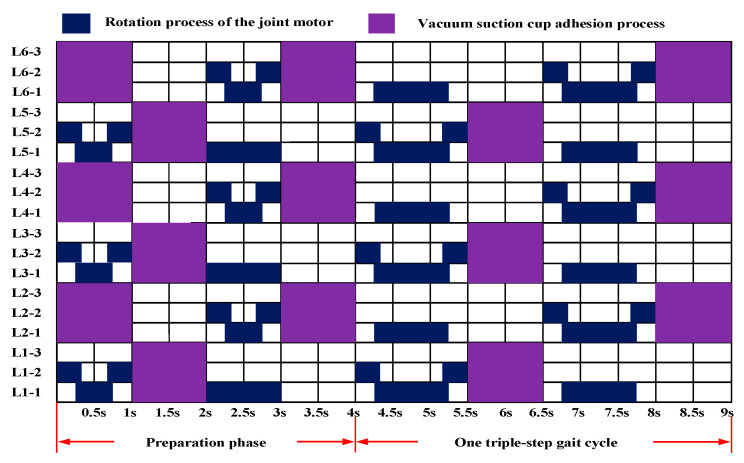
***Configuration I*** gait timing diagram.

During locomotion, the suction cups at the support leg ends must maintain continuous adhesion to the leaf surface. Consequently, as the basal segment rotates, the legs on both sides of the torso compress the torso. However, this compression can be passively accommodated through the coordination of the leg joints and ankle joints via passive ball-and-socket mechanisms. The joint motion sequence diagram for one gait cycle is shown in Figure 6.

**Figure 7:**
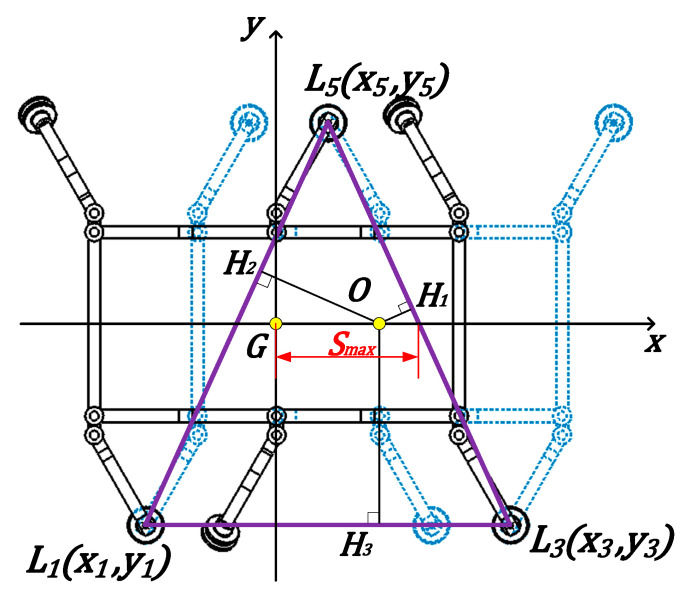
***Configuration I***: Gait Stability Margin. The black solid lines represent the robot’s footprint at the current time step, while the blue dashed lines represent the robot’s footprint at the next time step.

As shown in Figure 7, the black solid line represents the robot’s footprint, while the blue dashed line indicates the footprint for the next time step after the robot takes a step. ***O*** denotes the robot’s center of mass position, and (x_1_, y_1_), (x_3_, y_3_), and (x_5_, y_5_) are the foot landing coordinates for the robot’s first, third, and fifth legs, respectively. H_1_, H_2_, and H_3_ denote the distances from the center of mass to the three sides of the support polygon. ***GO*** represents the distance the robot moves forward, and ***Smax*** denotes the maximum stride length the robot can take while maintaining stability margin.

The maximum step length was determined using the static stability margin theory. First, the slope of ***L*_3_*L*_5_** was calculated using the coordinates of ***L*_3_** and ***L*_5_**:

(1) $$k_{L_{3}L_{5}}=\frac{y_{5}-y_{3}}{x_{5}-x_{3}}$$

The expression for the line segment ***L*_3_*L*_5_** is:

(2) $$y-y_{3}=\frac{y_{5}-y_{3}}{x_{5}-x_{3}}\left(x-x_{3}\right)$$

Since ***OH*_1_** is perpendicular to ***L*_3_*L*_5_**, the slope of ***OH*_1_** is:

(3) $$k_{OH_{1}}=\frac{x_{3}-x_{5}}{y_{3}-y_{5}}$$

The equation of the line ***OH*_1_** is:

(4) $$y=\frac{x_{3}-x_{5}}{y_{3}-y_{5}}x$$

The coordinates of the intersection point between ***OH*_1_** and ***L*_3_*L*_5_** are:

(5) $$\left(\frac{\left(y_{5}-y_{3}\right)\left(y_{5}x_{3}-x_{5}y_{1}\right)}{{\left(x_{3}-x_{5}\right)}^{2}+{\left(y_{3}-y_{5}\right)}^{2}},\frac{\left(x_{3}-x_{5}\right)\left(y_{5}x_{3}-x_{5}y_{3}\right)}{{\left(x_{3}-x_{5}\right)}^{2}+{\left(y_{3}-y_{5}\right)}^{2}}\right)$$

Similarly, the coordinates of ***H*_2_** and ***H*_3_** can be determined using the same computational steps as before, which will not be repeated here. Therefore, the distances from the center of mass to the boundaries of the supporting polygon are:

(6) $$OH_{1}=\frac{\left|y_{5}x_{3}-x_{5}y_{3}\right|}{\sqrt{{\left(x_{3}-x_{5}\right)}^{2}+{\left(y_{3}-y_{5}\right)}^{2}}}$$

(7) $$OH_{2}=\frac{\left|y_{1}x_{5}-x_{1}y_{5}\right|}{\sqrt{{\left(x_{5}-x_{1}\right)}^{2}+{\left(y_{5}-y_{1}\right)}^{2}}}$$

(8) $$OH_{3}=\frac{\left|y_{1}x_{3}-x_{1}y_{3}\right|}{\sqrt{{\left(x_{3}-x_{1}\right)}^{2}+{\left(y_{3}-y_{1}\right)}^{2}}}$$

During the calculation of static stability margin, it was determined that the displacement of the robot’s center of gravity in the forward direction must be confined between the two sides of the support triangle. Simultaneously, to ensure the center of gravity remains within the support triangle, the length for which stride S must be less than Smax is:

(9) $$S<S_{max}=x_{3}-\frac{x_{5}-x_{3}}{y_{5}-y_{3}}y_{3}$$

The gait configuration space designed for the climbing robot is 120°, corresponding to a stride length of 20 cm. This does not exceed the maximum stable stride length, thereby meeting the stability margin requirement.

### 3.2. Configuration II Gait Generation

**Figure 8:**
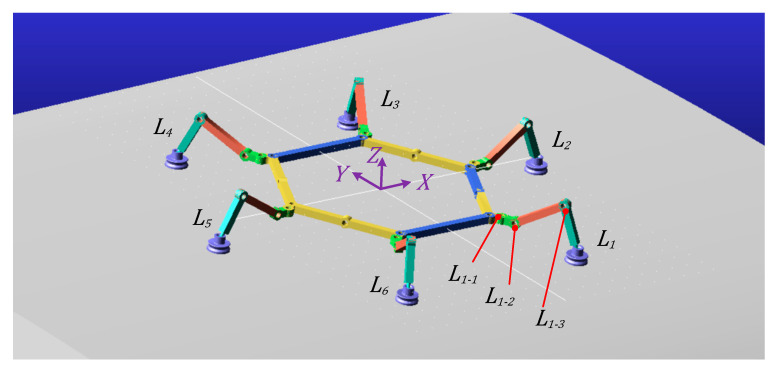
***Configuration II*** gait coordinate diagram.

The ***Configuration II*** triangular gait cycle requires completing the transition from ***Configuration I*** to ***Configuration II***, followed by a preparation phase before entering the triangular gait cycle. The initial state of ***Configuration II*** is shown in Figure 8. Initially, ***L*_2_**, ***L*_4_**, and ***L*_6_** remain in the support phase with the end-effector suction cups maintaining adhesion. ***L*_1_**, ***L*_3_**, and ***L*_5_** are in the swing phase: ***L*_1_** swings forward 60°, ***L*_3_** swings backward 30°, and ***L*_5_** swings forward 30°. ***L*_1_**, ***L*_3_**, and ***L*_5_** then transition to the support phase with suction cups adhering. Subsequently, ***L*_2_**, ***L*_4_**, and ***L*_6_** shift to the swing phase: ***L*_2_** swings forward 30°, ***L*_4_** swings backward 30°, and ***L*_6_** swings forward 60°. Concurrently, the basal segments of ***L*_1_**, ***L*_3_**, and ***L*_5_** rotate 60°, propelling the main body forward by one triangular gait stride distance. Thereafter, the triangular gait cycle commences, with ***L*_1_**, ***L*_3_**, and ***L*_5_** alternating with ***L*_2_**, ***L*_4_**, and ***L*_6_** between support and swing phases to propel the torso forward.

**Figure 9:**
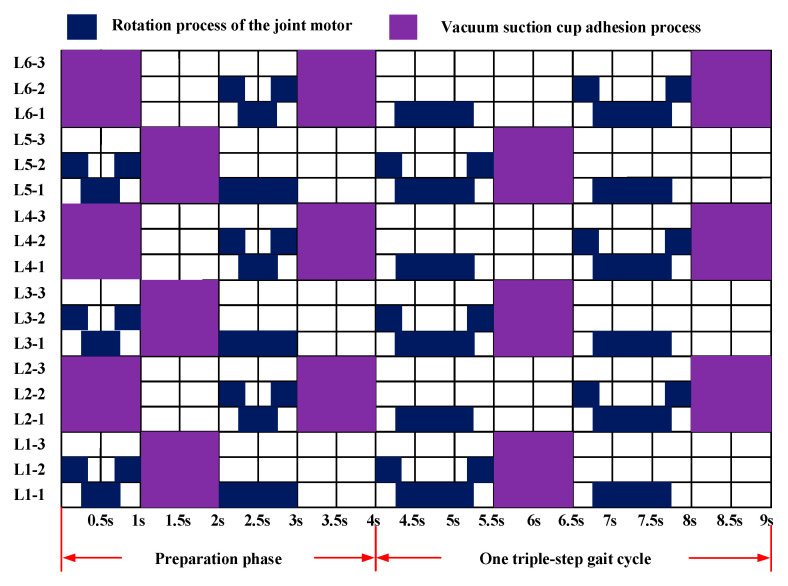
Gait timing diagram for ***Configuration II*** perpendicular to the trunk edge direction.

In ***Configuration II***, the triangular gait remains a relatively efficient and stable gait pattern; therefore, it continues to serve as the basis for analysis. Figure 9 illustrates the gait timing diagram for ***Configuration II*** when the gait direction is perpendicular to the side length of the torso.

**Figure 10:**
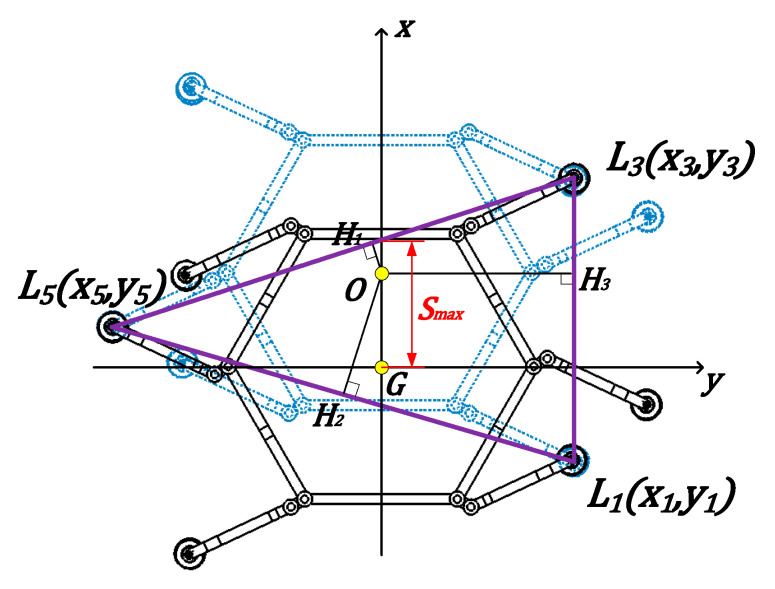
Stability margin of gait in ***Configuration II*** perpendicular to the long axis of the trunk. The black solid lines represent the robot’s footprint at the current time step, while the blue dashed lines represent the robot’s footprint at the next time step.

In ***Configuration II***, when the robot adopts a gait perpendicular to the edge length of its torso, the method for calculating its static stability margin remains similar to previous approaches. Walking stability is still assessed by determining the distance between the real-time center of mass position and each side of the support polygon. As shown in Figure 10, the black solid line represents the robot’s footprint, while the blue dashed line indicates the footprint for the next time step after the robot takes a step. ***O*** denotes the robot’s center of mass position, and (x_1_, y_1_), (x_3_, y_3_), and (x_5_, y_5_) represent the landing point coordinates of the robot’s first, third, and fifth legs, respectively. H_1_, H_2_, and H_3_ denote the distances from the center of mass to the three sides of the support polygon. ***GO*** represents the robot’s displacement in the forward direction, while Smax indicates the maximum stride length the robot can take while maintaining a stability margin.

The calculation method for the maximum stride length within the stability margin is identical to that of ***Configuration I*** and will not be repeated here. To ensure the center of gravity remains within the support triangle at all times, the stride length ***S*** must be less than ***Smax***. The gait space designed for the climbing robot is 60°, corresponding to a stride length of 20 cm. This value does not exceed the maximum stable stride length, thus satisfying the stability margin requirement.

## 4. Simulation Analysis

### 4.1. Configuration I Gait Simulation Analysis

**Figure 11:**
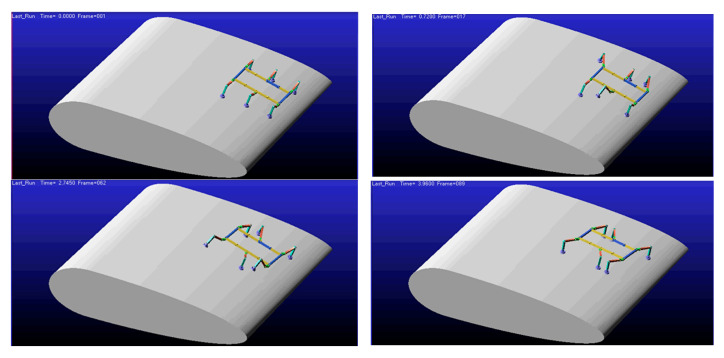

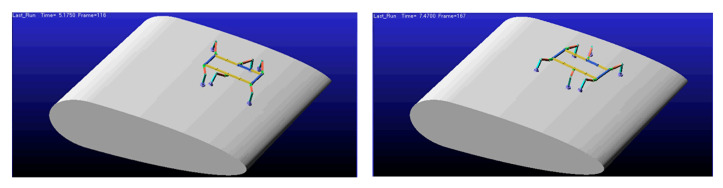
***Configuration I***: Gait Simulation Diagram.

In Adams, import the simplified robot model and blade model. Set the coordinate origin at the geometric center of the robot’s torso. The ***X-axis*** aligns with the torso’s length and points forward. The ***Y-axis*** aligns with the torso’s width. with the ***Z-axis*** perpendicular to the fuselage and pointing upward. Configure the link types and drives, set the contact modes for the six leg endpoints with the blades, and perform a full gait cycle simulation using triangular gait parameters. The states at each phase are shown in Figure 11. Additionally, Supplementary Video S1 provides a complete dynamic demonstration of this gait pattern.

**Figure 12:**
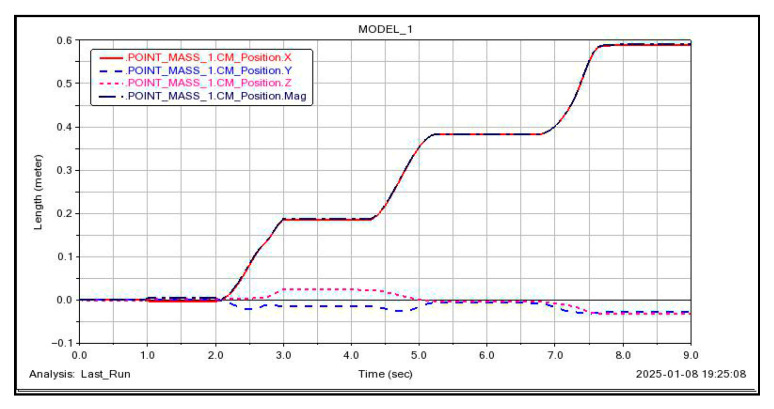
***Configuration I***: Center of Gravity Changes During Gait. The impact of ***Y*** and ***Z*** displacements on overall accuracy is negligible, although their fluctuation range is less than 3% of the ***X*** displacement.

A relationship showing the change in the robot’s center of gravity position over time was generated, as illustrated in Figure 12. The curvature of blade surface A changes relatively gradually, with the forward direction of the body nearly aligned with the ***X-axis***. During one triangular gait cycle, the model moves 20 cm along the ***X-axis***, with maximum displacements of 2.5 cm along both the ***Y-axis*** and ***Z-axis***. This occurs because during forward motion, the fixed suction cups at the end effector cause the body to be compressed as the legs swing on either side. This process is passively regulated by the spherical joints at the ankle joints of the legs. Consequently, fluctuations in the ***Y*** and ***Z*** directions occur, but these fluctuations are relatively small and negligible. Moreover, these fluctuations cancel each other out after two triangular gait cycles.

### 4.2. Configuration II Gait Simulation Analysis

**Figure 13:**
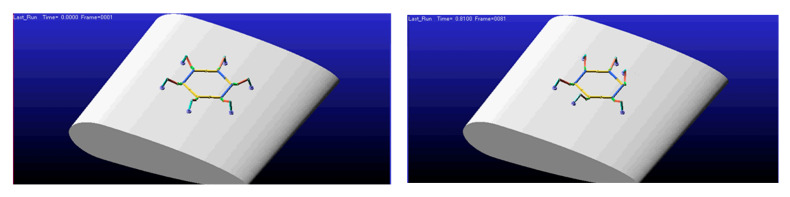

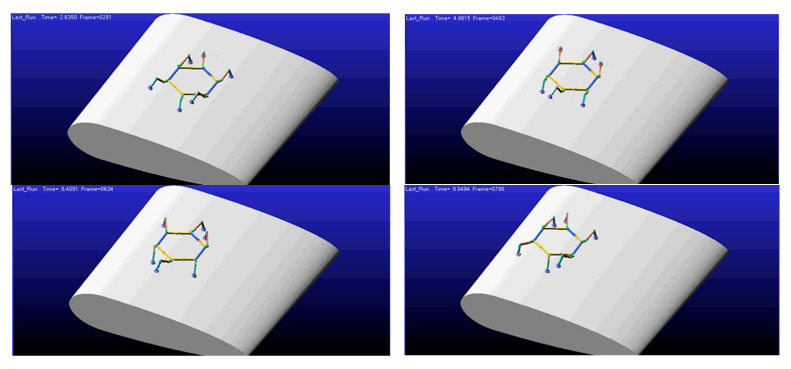
***Configuration II***: Gait Simulation Diagram.

In Adams, import the simplified robot model and blade model. Similar to the ***configuration I*** triangular gait simulation, set the coordinate origin at the geometric center of the robot’s torso. The ***X-axis*** aligns with the torso’s length direction and points forward. The ***Y-axis*** aligns with the torso’s width direction. The ***Z-axis*** is perpendicular to the body and points upward. Configure the joint types and actuation. Set the contact modes for the six leg endpoints with the blades. Perform a full gait cycle simulation using the triangular gait parameters. The states for each phase are shown in Figure 13. Additionally, Supplementary Video S2 provides a complete dynamic demonstration of this gait pattern.

**Figure 14:**
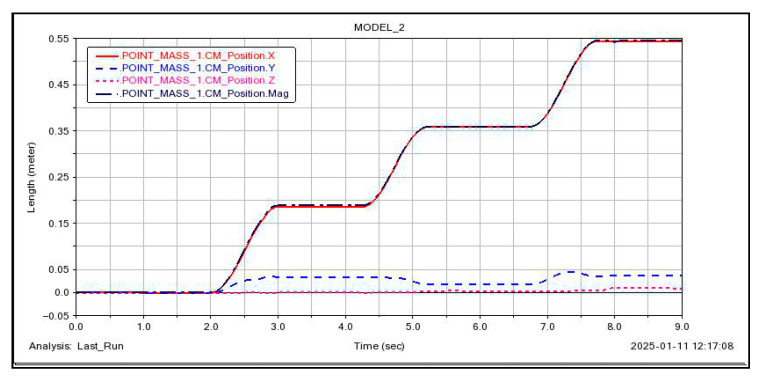
***Configuration II***: Center of Gravity Changes During Gait. The impact of ***Y*** and ***Z*** displacements on overall accuracy is negligible, although their fluctuation range is less than 3% of the ***X*** displacement.

A relationship showing the change in the robot’s center of gravity position over time was generated, as illustrated in Figure 14. In configuration two, perpendicular to the edge length direction of the torso, the robot completes one triangular gait cycle. The model moves 20 cm along the ***X-axis***, with a maximum displacement of 5 cm along the ***Y-axis*** and 1.5 cm along the ***Z-axis***. The fluctuations in the ***Y*** and ***Z*** directions are relatively small and negligible, and these fluctuations cancel each other out after two triangular gait cycles.

## 5. Prototype Validation

To validate the effectiveness of the designed gait planning method, we experimentally verified the robot’s load-bearing capability and gait stability under various configurations. Experimental results demonstrate that the robot’s motion performance across different configurations aligns closely with theoretical analysis, proving the applicability of the gait planning strategy on complex wind turbine blade topographies.

### 5.1. Load Adsorption Experiment

**Figure 15:**
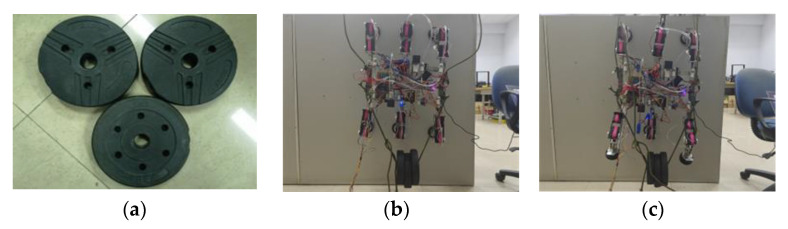
Robot load adhesion test: (**a**) Counterweight total: 6 kg; (**b**) Six-legged adhesion load: 6 kg; (**c**) Three-legged adhesion load: 6 kg.

This wind turbine blade climbing robot is designed with a payload capacity of 5 kg. The prototype underwent load testing with a 6 kg payload, aligning with practical operational requirements while maintaining adequate load margin. As shown in Figure 15, the prototype demonstrated stable adhesion on vertical surfaces in both six-legged and three-legged suction configurations. Vacuum pump pressure was maintained at 88 kPa, with no suction cup detachment observed. The adhesion reliability meets design specifications.

### 5.2. Configuration I Gait Experiment

**Figure 16:**
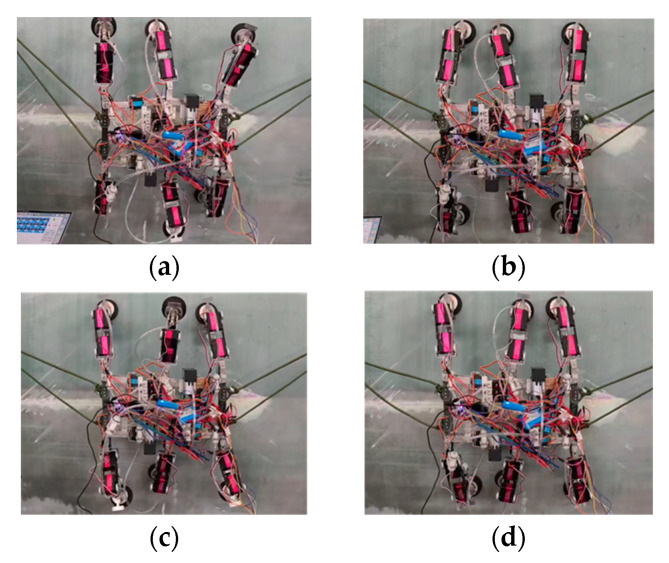
Robot gait experiments under ***Configuration I***: (**a**) Leg groups 1, 3, and 5 lift and move forward one stride; (**b**) Leg groups 1, 3, and 5 transition to the support phase; (**c**) Leg groups 2, 4, and 6 lift and move forward one stride; (**d**) Leg groups 2, 4, and 6 transition to the support phase.

On the surface of a wind turbine blade with a curvature radius of 1.5 m, the robot performs climbing experiments using a triangular gait in the planar quadrilateral configuration of ***Configuration I***. The process is shown in Figure 16: the vacuum suction cups at the ends of the six legs adhere to the surface, the torso remains horizontal, and the angle between the basal segments of the legs is 90°. Through controller commands, legs 1, 3, and 5 were raised and swung 60°, while the base segment servos of the supporting leg groups rotated to advance the torso 20 cm. After the swinging leg groups landed, they transitioned to the support phase, repeating the gait cycle. During the forward movement of the robot’s center of gravity, no significant instability was observed, demonstrating excellent gait stability. The vacuum pump pressure was maintained at 88 kPa, and the suction cups did not detach.

### 5.3. Configuration II Gait Experiment

**Figure 17:**
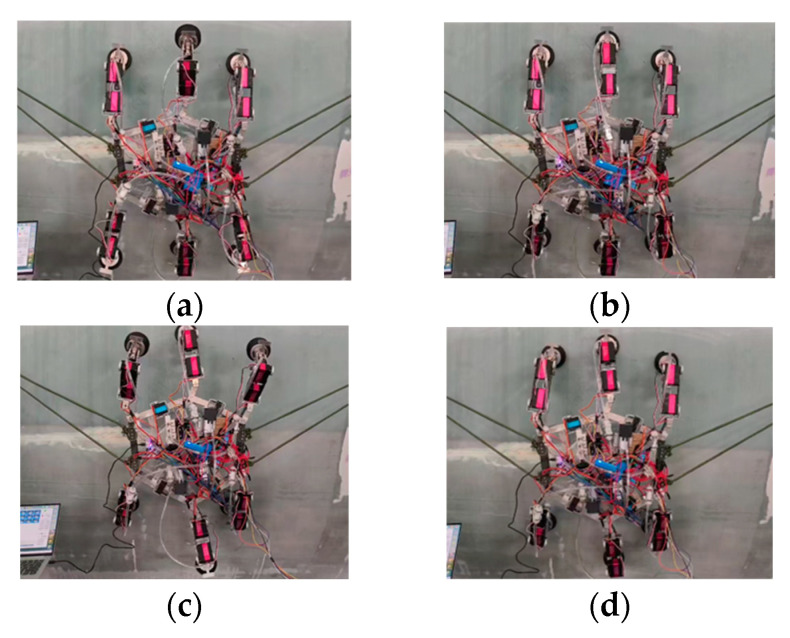
***Configuration II*** vertical edge length direction gait experiment: (**a**) 1-3-5 leg group lifts and moves forward one stride; (**b**) 1-3-5 leg group transitions to support phase; (**c**) 2-4-6 leg group lifts and moves forward one stride; (**d**) 2-4-6 leg group transitions to support phase.

In ***Configuration II’s*** regular hexagonal form, the robot tested its gait process perpendicular to the body’s edge length, as shown in Figure 17. The six-legged base segment servos rotated in coordination, driving the robot to move along a direction perpendicular to the hexagon’s edge length. The measured single stride length was 18 cm. During the forward movement of the robot’s center of gravity, no significant instability was observed, demonstrating excellent gait stability. Vacuum pump pressure was maintained at 88 kPa, with no suction cup detachment observed.

## 6. Discussion

The theoretical core of this study lies in integrating variable-cell kinematics with robotic gait planning, providing a solution based on discrete topological reconstruction for mobility problems on unstructured surfaces. The robot’s planar quadrilateral and regular hexagonal configurations are essentially two parallel mechanism configurations exhibited by its closed-loop folded torso under different kinematic constraints. The configuration switching process constitutes a controlled mechanism reconstruction motion, whose successful execution relies on precise planning of multi-leg coordination and the trajectory of the system’s center of gravity within the reconstruction space—transcending the scope of traditional fixed-configuration robot gait planning. This study systematically applies static stability margin theory to such topologically variable robots, revealing that their stable gait space is directly constrained by the geometric shape of the instantaneous support polygon. The theoretically derived maximum stable stride length provides a universal quantitative criterion for gait parameter optimization, ensuring the intrinsic stability of the robot’s motion across all configurations.

Simulations and experiments validate the effectiveness of this theoretical framework. The precise tracking of the robot’s center-of-mass trajectory along the forward direction, coupled with small periodic oscillations in lateral and normal directions, demonstrates that the planned gaits not only satisfy static stability conditions but also exhibit excellent kinematic consistency. Future theoretical development should focus on establishing dynamic stability criteria for configuration transitions and exploring online autonomous reconfiguration planning coupled with real-time environmental perception. This will further refine the theoretical framework for applying reconfigurable mobile robotics in complex environments.

This study currently relies primarily on assumptions of static stability and idealized environments, presenting several limitations for practical application: Regarding surface adaptability, current experiments are conducted on dry, rigid surfaces without fully accounting for the impact of wet or oily terrain on foot adhesion, factors that may reduce the effectiveness of support polygons; Regarding environmental disturbances, the impact of external perturbations like wind and vibration on lightweight folding structures remains unquantified, potentially challenging the static stability assumption. Additionally, dynamic factors are simplified during rapid reconfiguration; inertial forces and impacts during actual transitions may cause transient stability issues. Finally, concerning perception dependency, this study employs offline planning under the assumption of known static environmental information, without integrating real-time sensor feedback to address dynamic obstacles.

To address these limitations, future work will focus on: establishing dynamic stability criteria to investigate the transient dynamics during configuration switching and impact mitigation strategies; developing online environmental perception and autonomous reconfiguration algorithms that integrate visual/tactile feedback for real-time topological decision-making on unknown terrain; studying environmental adaptability by quantifying the impact of factors like humidity and surface roughness on traction, and designing fault-tolerant gaits; exploring energy-optimized planning to balance reconfiguration frequency and motion efficiency. These extensions will enhance the robustness of this theoretical framework in real-world complex scenarios.

## 7. Conclusions

This paper designs a wind turbine blade climbing robot featuring a 10R folding variable-cell closed-loop configuration. It adapts to varying surface curvatures across different blade segments through two reconfigurable morphologies: a planar quadrilateral and a regular hexagon. The study elucidates the topological conditions for configuration switching and the multi-leg cooperative control mechanism. Based on static stability margin theory, the maximum stable stride length for both configurations is determined to be 20 cm. Simulation analysis further validates the kinematic feasibility of multiple gait strategies. Prototype experiments demonstrate excellent climbing stability under different configurations on simulated blades, effectively verifying the proposed mechanism design and gait planning methods.

Despite these advances, the study has limitations: current experiments rely on dry, stable simulated environments and do not fully account for the potential impact of real-world conditions like wetness, oil contamination, or wind-blown sand on adhesion and load-bearing capacity. The coupling effects between configuration switching dynamics and external disturbances require further quantification. Additionally, the existing system depends on offline planning and lacks real-time environmental perception capabilities. Furthermore, energy optimization and durability during prolonged climbing operations require further evaluation.

Future work will address these limitations by carrying out the following: integrating visual and force sensing to develop online environmental perception and autonomous navigation algorithms, enabling intelligent reconfiguration decisions under unknown curvatures and obstacles; conducting field experiments on real blades to systematically validate operational reliability in extreme conditions such as humidity, low temperatures, and variable wind speeds; and establishing dynamic stability criteria to optimize impact suppression and energy efficiency during switching processes. The ultimate goal is to advance this robot toward practical engineering applications in wind turbine maintenance, thereby enhancing the level of intelligent maintenance in wind power.

## Figures and Tables

**Table 1:** Wind Turbine Blade Climbing Robot Joint Function Assembly.

Joint Designation	Joint Name	Joint Type	Function Description
***A, E, F***	Corner joint	Active Servo Joint	The drive mechanism transforms the trunk from a rectangular to a regular hexagonal configuration, with a rotation range of 0–120°.
***B, D, G, I***	Double-ended swing joint	Active Servo Joint	Control the front and rear ends of the body to bend vertically and horizontally from 0 to 90 degrees, enabling it to traverse over the leading or trailing edge of a blade.
***J***	Passive joint rotation	Passive joint	Connect carbon fiber plates to adaptively follow trunk deformation.
***C, H***	Motor Connectors	structural components	Connect the motor to the carbon fiber plate to transmit torque.
***L*_1*-*1_ *, L*_3*-*1_ *, L*_4*-*1_ *, L*_6*-*1_**	Clamp the femoral base joint	Active Servo Joint	Drive the clamping leg to swing forward and backward, enabling adjustment within a ±30° range.
***L*_2*-*1_ *, L*_5*-*1_**	Weight-bearing leg basal joint	Active Servo Joint	Drive the load-bearing legs to swing forward and backward, enabling adjustment within a ±30° range.
***L_X-_*_2_** (X = 1~6)	Hock joint	Active Servo Joint	Drive the hip joint to swing within the leg plane, adjusting the leg posture.
***L_X-_*_3_** (X = 1~6)	tibial joint	Active Servo Joint	Drive the tibial segment to swing within the leg plane, coordinating with the foot end to conform to the surface.

## Data Availability

The original contributions presented in this study are included in the article. Further inquiries can be directed to the corresponding author.

## Supplementary Materials

The following supporting information can be downloaded at: https://www.mdpi.com/article/10.3390/s26020547/s1, Video S1: Configuration I Gait Simulation Animation; S2: Configuration II Gait Simulation Animation; As dynamic visual evidence for the gait planning algorithm described in the main text, we simultaneously submit two simulation videos created using Adams. These videos vividly recreate the robot’s walking process under two different configuration parameter sets, providing an intuitive comparison that validates the analysis conclusions regarding gait stability and maneuverability presented in Section 3.1 and Section 3.2 of the main text. Please note that the supplementary videos demonstrate a simplified contact simulation—actual adhesion has been experimentally verified.
